# Comparative Study of Malaria Prevalence among Travellers in Nigeria (West Africa) Using Slide Microscopy and a Rapid Diagnosis Test

**DOI:** 10.1155/2015/108707

**Published:** 2015-05-12

**Authors:** T. V. Dougnon, H. S. Bankole, Y. M. G. Hounmanou, S. Echebiri, P. Atchade, J. Mohammed

**Affiliations:** ^1^Research Laboratory in Applied Biology, Polytechnic School of Abomey-Calavi, University of Abomey-Calavi, 01 BP 2009 Cotonou, Benin; ^2^Department of Biotechnology, African University of Technology and Management, Campus of Gbegamey, 04 BP 1361 Cotonou, Benin; ^3^Department of Veterinary Microbiology and Parasitology, Sokoine University of Agriculture, P.O. Box 3051, Chuo Kikuu, Morogoro, Tanzania

## Abstract

Malaria is a major disease in Africa and leads to various public health problems. A study was carried out at the Aviation Medical Clinic Laboratory, Murtala Mohammed Airport, Ikeja, Lagos State, Nigeria, in 2014. The work aimed to determine the prevalence of malaria among patients attending the laboratory. Blood samples were therefore collected from 51 patients and subjected to both blood smear microscopy and a rapid immunochromatographic diagnostic test (SD BIOLINE Malaria Ag) for detection of, respectively, malaria parasites and antigens. At the end of the study, 22% of the patients were detected positive by the microscopic examination while 9.8% were tested positive when using SD BIOLINE Malaria Ag. The outcomes of the study show a high prevalence of malaria at the airport. This represents a serious risk factor leading to a high likelihood of spread and occurrence of malaria in other countries including Western countries whereby the disease is nonendemic. It also pointed out that the blood smear microscopy seems to be better than Rapid Diagnosis Test (RDT) for malaria diagnosis.

## 1. Introduction

Malaria is one of the most common infectious diseases and a great public health problem worldwide. It is one of the world's deadliest diseases affecting people particularly in tropical and subtropical regions of the world, especially in sub-Saharan Africa and Southeast Asia.

Malaria's mortality rates have reached 42% globally since 2000 and 49% in the African region [1]. Of the estimated malaria deaths in 2012, more than 80% occurred in 18 African countries with Democratic Republic of Congo and Nigeria together accounting for 40% of the estimated global count [2]. These areas are therefore considered as hyperendemic areas as far as malaria is concerned. Moreover, malaria constitutes a very dangerous disease for word travellers especially to those from nonendemic malaria countries. For instance, studies conducted by Stauffer et al. [3] showed that over 1500 cases of malaria are annually reported in the United States on travellers who return to their country. Because malaria is unusual in the United States, patients present to primary health care facilities that lack expert diagnostic capabilities with no tropical medicine expertise. This might therefore be fatal if appropriate diagnostic is not carried out in the visiting countries' airport before they return back. For efficient malaria control, early diagnosis in sick patients is required for prompt treatment to everyone and to foreign travellers in particular. Therefore, adequate diagnosis tests are needed at the health care facilities. However, the standard blood smear microscopic examination that provides the most comprehensive information on a single test format, being the “gold standard” for the diagnosis of malaria, is not available at all primary aviation health care levels in many malaria endemic areas. Accordingly, alternative approaches have been sought. Nevertheless, their cost effectiveness and accuracy remain questionable. In this study the performance of a Rapid Diagnostic Test was assessed and compared to the microscopic test on patients attending the Aviation Medical Clinic Laboratory, Murtala Mohammed Airport, Ikeja, Lagos State, Nigeria. The RDT used was SD BIOLINE Malaria Antigen (*Pf*-*p*LDH and/or pan-*p*LDH), a commercial immunochromatographic test based on plasmodium species-specific antigens that allow differential detection of* P. falciparum* and other species (*Plasmodium vivax, Plasmodium ovale, *and* Plasmodium malariae*). It is the commonly used and the one available at this health care centre.

## 2. Materials and Methods

### 2.1. Study Area

This study was carried out in the Aviation Medical Clinics, which is situated within the Murtala Mohammed International Airport, Ikeja, Lagos, Nigeria, in 2014. In order to achieve the objective of this study which was to investigate the possibilities of malaria transportation from Nigeria to other countries via travellers, this aviation clinic was chosen. It is under the Medical Department of the Federal Airports Authority of Nigeria (FAAN). It provides medical facilities to staff of FAAN, Nigeria Airspace Management Authority (NAMA), and Accident Investigation and Protection Bureau (AIB). It also responds to emergencies involving passengers and other airport users.

### 2.2. Study Population

Patients who visited the clinic during the study period with malaria symptoms were recruited for this study regardless of their age and sex. Participants were children (4-5 years old), adolescents (6–17 years), and adults (≥18 years) with severe and uncomplicated malaria. These samples were stratified by age so as to be able to assess the relationship of age with these parameters. Regarding ethical clearance, approval from the management of Aviation Medical Clinic was obtained before the implementation of the study. Moreover, an informed consent form was obtained from subjects participating in the study. For children <15 years, the consent was obtained from their parents.

### 2.3. Sample Collection and Processing

Venous blood was collected using 5 mL plastic syringe attached to a 20 SWG needle. In case the patient's veins are small a 23 SWG needle was used. Blood was collected in tubes containing ethylene diamine tetra acetic acid (EDTA). For finger prick, blood was collected on slides using the needle of a stylet from the fingertip of participants. Thick and thin blood films of the blood samples were made and air dried. Thin films were fixed with methanol and both thick and thin films were then stained with Giemsa. The slides were microscopically analysed for the presence of species of malaria parasites.

The blood samples collected by finger prick were assayed using SD BIOLINE Malaria Ag test kits. It is an immunochromatographic test that detects the presence of pan malaria specific antigen (pLDH: plasmodiumlactate dehydrogenase) for the detection of all malarial parasites.

The test uses approximately 5 *μ*L of blood and is readable after 15 minutes following the manufacturer's instructions (Standard Diagnostics, Republic of Korea). The results were recorded in comparison with the control line as positive if a unique* Pf*-*p*LDH line appears indicating* P. falciparum* infection and/or pan-*p*LDH line indicating either an infection with* P. falciparum* or a mixed infection with* P. falciparum* and one or more of the* nonfalciparum* species.

### 2.4. Statistical Analysis

Data were entered in Microsoft Excel 2013. Distribution of participants' characteristics and malaria parasite prevalence were assessed using contingency tables. Taking blood slide microscopy as the gold standard, the performance of the Rapid Diagnostic Test was compared to it by computing the sensitivity, specificity, the negative predictive value, and the positive predictive value of the test as per Wayne [4].

## 3. Results

### 3.1. Characteristics of Study Population

Fifty-one persons of both sexes from 4 to 73 years old were recruited for the study (Table 1). The group was composed of 23 males and 28 females distributed as follows: 2 infants, 8 adolescents, and 41 adults. 

**Figure 1:**
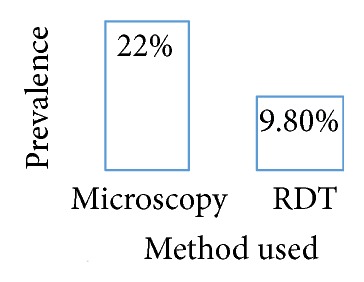
Prevalence of malaria at Aviation Medical Clinic, Murtala Mohammed Airport, Ikeja, Lagos State, Nigeria.

**Figure 2:**
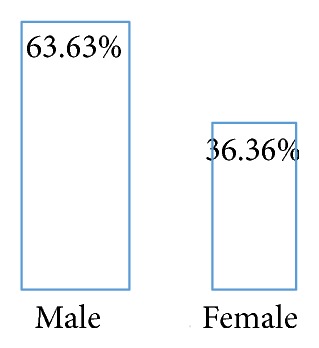
Prevalence of malaria according to patients' gender using microscopy.

Out of the 51 persons recruited for this study, 11 patients were tested positive for malaria in microscopic examination, while 5 samples were tested positive with the RDT. The actual prevalence of malaria among patients attending this clinic is therefore about 22% using microscopy and 9.8% in the case of RDT (Figure 1). Furthermore, four of the 11 microscopically positive cases were females and the other seven were males. There is therefore a sexual distribution in the malaria positive results given by the microscopic examination whereby males seem to be more infested by the malaria parasite (63.33%) than females (36.36%) (Figure 2).

### 3.2. Performances of the RDT Used

The RDT used in this study is for malaria's antigen detection in order to assess its effectiveness by comparing it to the gold standard. However, only 5 out of the 11 positives (microscopy) were tested positive for* P. falciparum* using RDT.

Table 2 is the contingency table obtained from the results.

From Table 2, Table 3 was drawn to establish the performances of the test. This table displays the sensitivity (45.45%) and the specificity (100%) together with the positive predictive value (100%) and the negative predictive value (86.9%) of the RDT used in this study. Furthermore, the RDT of this study showed a very high specificity and lower sensitivity. This test seems to detect more true negative cases than the microscopy's method. As a matter of comparison, RDT has the ability to detect more negative cases and less positive cases than microscopic test does.

## 4. Discussion

Malaria prevalence detected by slide microscopy in this study was about 22%. This prevalence is relatively high but normal because of the endemicity of malaria in the study area. However, it shows a threat of malaria spread from Nigerian to other foreign airport users who might probably be coming from nonendemic malaria countries. As a matter of fact, Medina Costa et al. [5] reported that about 40.29% of travellers with a possible history of malaria exposure were positive for anti-*Plasmodium *spp. antibodies. Such situation can therefore compromise the health safety of Western countries' travellers in case they are not diagnosed and do not receive adequate malaria treatment before going back to their home countries, whereby they may not have the chance to receive specific malaria treatment. Nevertheless, RDT detected only 9.8% of malaria positive cases at the same facility on the same sample types. According to previous comparative studies between RDT and microscopic malaria diagnostic tests, RDT usually provides greater prevalence than microscopy [6, 7]. With regard to this, Hendriksen et al. [7] concluded that RDT is an acceptable alternative to routine microscopy for diagnosing severe malaria cases. However, the low prevalence of malaria found by the RDT compared to the slide microscopy in this study has been also established by Endeshaw et al. [8]. In fact, the difference could be due to the type of antigen targeted by the RDT used in these studies.

For instance, most of studies that came out with higher prevalence from RDT than microscopy are based on* Plasmodium falciparum *specific histidine rich protein-2 (HRP-2) [6], while in the current study as well as studies conducted by Endeshaw et al. [8] the RDTs used were based on* Pf*-*p*LDH. The difference between these two types of antigens targeted during the RDT that could lead to differences in the detection proportion was previously explained by Murray et al. [9]. They demonstrated that the HRP-2 assay showed greater sensitivity compared to the pLDH antigen based assay. Similar scenario was reported as well by Kocharekar et al. [10]. According to Murray et al. [9], the HRP-2 antigen is known to possibly persist at detectable levels for more than 30 days, after the symptoms have disappeared, and the forms that cause disease have been cleared from the patient's blood, while RDTs based on* Pf*-*p*LDH rapidly fall to undetectable levels after initiation of effective therapy. Likewise, Kocharekar et al. [10] testified this idea in their experimentation in malaria patients. In fact, at the beginning of their experiment (being at the early stages of the disease) the* Pf*-*p*LDH based RDT was giving higher sensitivity (95%) than HRP-2 based RDT (70.3%). Fifteen days later, while patients were undertaking treatment, the sensitivity of* Pf*-*p*LDH based RDT falls to 7.92%, while the one of HRP-2 based RDT still gave as high sensitivity as 58.41%. In other words, positive cases detected by the PfHRP2 based test can be regarded as false positive cases since people do not normally have the parasite in their blood stream anymore. Furthermore, considering that some of the patients used in this study might be undergoing malaria treatment before the sampling has been done, the RDTbased on* Pf-p*LDH might fall to signal them as positive. This justifies the low prevalence of malaria given by the RDT in this study compared to the slide microscopy. Moreover, there was a sexual distribution of the prevalence, showing that male patients were more infected by malaria parasite than female patients. This suggests that males may probably be at higher risk of mosquito bites than females, which could be explained by the fact that African women sleep under mosquito nets freely received at health care centres during pregnancy and for their children protection.

Moreover, the sensitivity of RDT in this study was low (45.45%). The probability of RDT to detect positive cases among microscopically positive cases is therefore much less. This low sensitivity can be due to either the very low parasitaemia in people not seeking treatment or the fact that the RDTs were possibly defective or handled inappropriately causing them to lose sensitivity.

Factors like products, storage temperature, and humidity can affect the diagnostic accuracy [8]. However, the specificity of the current RDT was very high (100%), meaning that the probability of RDT to detect negative cases among microscopically negative cases is as high as 100%. This test is thus very good to have confidence in the positive results since it detects at 100% all negative cases who are actually nondiseased, helping therefore to give treatment to the appropriate diseased people only. Besides, the positive predictive value of the RDT (proportion of individuals with positive RDT results among those who were malaria positive according to the microscopy) was 100%, while the negative predictive value (proportion of individuals with negative RDT results among those who were malaria negative according to the microscopy) was 86.95%. The clinical implication of the high PPV (100%) of this RDT is that any individual who has a positive RDT screening test would have high probability of having malaria with respect to slide microscopy. Though predictive values are known to strongly depend on disease prevalence in a given population, they are more clinically useful than the specificity and the sensitivity of a test because they give practical usefulness information on the test. In the case of this study, any individual who will be tested at any hospital or laboratory with a positive result using this RDT is highly likely to be malaria positive. In such condition, every positive result should be considered as true case with no doubt or uncertainty and therefore be treated immediately. It shows that doctors should treat only people who are actually truly positive and avoid treating wrongly a false positive patient. If it is not done like that, it can lead to emergence of antimalarial resistance.

## 5. Conclusion

Out of this study, it has been noticed that the prevalence of malaria among people around airports is not negligible and must be further investigated since these places are like interface between foreign people and natives. They are also considered as a risk factor for spread of malaria in developed countries. Highly sensitive and specific diagnostic tools are therefore needed in these areas. Besides, based on the results of this study, well-conducted blood slide microscopy for malaria diagnosis remains the preferred option. The low level of the agreement between RDT and slide microscopy for malaria diagnosis warrants further investigations in clinical facilities and aviation clinic laboratories especially.

## Figures and Tables

**Table 1:** Age distribution of people enrolled in the study.

	Age groups (years)			Total
	4-5	6–17	≥18	
Gender				
Male	1	4	18	23
Female	1	4	23	28
Total	2	8	41	51

**Table 2:** 2 × 2 contingency table for assessing the RDT's performance.

	Microscopy +	Microscopy −	Total
RDT+	5	0	5
RDT−	6	40	46
Total	11	40	51

**Table 3:** Accuracy parameters of the RDT using the slide microscopy as gold standard.

Accuracy measures	Values
Sensitivity	45.45%
Specificity	100%
Positive predictive value	100%
Negative predictive value	86.95%
